# Interlaboratory comparison of high-throughput protein biomarker assay quantifications for radiation exposure classification

**DOI:** 10.1371/journal.pone.0301418

**Published:** 2024-04-29

**Authors:** Leah Nemzow, Thomas Boehringer, Jessica Mayenburg, Lindsay A. Beaton-Green, Ruth C. Wilkins, Helen C. Turner

**Affiliations:** 1 Center for Radiological Research, Columbia University Irving Medical Center, New York, New York, United States of America; 2 Consumer and Clinical Radiation Protection Bureau, Health Canada, Ottawa, Ontario, Canada; Mustansiriyah University, IRAQ

## Abstract

In the event of a widespread radiological incident, thousands of individuals will require rapid assessment of exposure using validated biodosimetry assays to inform clinical triage. In this scenario, multiple biodosimetry laboratories may be necessary for large-volume sample processing. To meet this need, we have developed a high-throughput assay for the rapid measurement of intracellular protein biomarkers in human peripheral blood samples using an Imaging Flow Cytometry (IFC) platform. The objective of this work was to harmonize and validate the reproducibility of our blood biomarker assay for radiation exposure across three IFC instruments, two located at Columbia University (CU) and the third at Health Canada. The Center for Radiological Research (CRR) at CU served as the central laboratory and reference instrument, where samples were prepared in triplicate, labeled with two radiation responsive leukocyte biomarkers (BAX and phosphor-p53 (Ser37)), and distributed for simultaneous interrogation by each IFC. Initial tests showed that significantly different baseline biomarker measurements were generated on each instrument when using the same acquisition settings, suggesting that harmonization of signal intensities is necessary. Subsequent tests harmonized biomarker measurements after irradiation by modulating laser intensity using two reference materials: unstained samples and standardized rainbow beads. Both methods generated measurements on each instrument without significant differences between the new and references instruments, allowing for the use of one master template to quantify biomarker expression across multiple instruments. Deming regression analyses of 0–5 Gy dose-response curves showed overall good correlation of BAX and p53 values across new and reference instruments. While Bland-Altman analyses indicated low to moderate instrument biases, ROC Curve analyses ultimately show successful discrimination between exposed and unexposed samples on each instrument (AUC values > 0.85).

## Introduction

In the event of a large-scale radiological emergency, such as the detonation of an improvised nuclear device (IND) or meltdown of a nuclear reactor, potentially thousands of people will need to quickly be assessed for radiation exposure. To reduce strain on limited medical supplies and facilities, an effective early triaging strategy will be important in guiding medical treatment [1]. Quickly relieving concerned citizens who do not require medical intervention will be an especially critical part of managing resources [2,3]. Radiation biodosimetry assays capable of rapidly estimating levels of exposure are a significant component of emergency response, yet no United States Food and Drug Administration (FDA)-cleared assays currently exist [1,4–6]. To this end, we have developed a high-throughput, Imaging Flow Cytometry (IFC)-based blood test to rapidly quantify levels of intracellular protein biomarkers for radiation dose estimation [7,8]. Despite the speed and sample capacity of this assay, multiple IFC instruments will be necessary to overcome the surge in sample quantities in a mass casualty scenario. Performance validation of these biomarkers on instruments located at different sites is an important step towards technological readiness of this bioassay for FDA-cleared use in a mass-casualty scenario.

The objective of this work was to validate and harmonize the reproducibility of our blood biomarker quantifications and radiation exposure classifications on three ImageStreamX (ISX) MkII IFC instruments: “CU-Reference”, “CU-FlowCore”, (both located at Columbia University) and “HealthCanada”. Towards this goal, we designed an interlaboratory study to test the variability between sample measurements from each instrument, as well as to develop methods for generating biomarker responses within acceptable limits of agreement on each instrument for radiation discrimination. In this study, our laboratory at the Center for Radiological Research (CRR) served as the reference laboratory: at CRR, whole blood samples were X-irradiated and cultured *ex vivo*, fixed and stained in triplicate with our top two performing biomarkers, BAX and phosphor-p53 (Ser37) [8], and subsequently distributed for simultaneous acquisition on the other two IFC instruments.

Here, we present two methods for harmonizing fluorescence intensity measurements on ISX MkII instruments, and we evaluate the reproducibility of the biomarker quantifications by comparing coefficient of variation (%CV) values, correlation, bias, and limits of agreement on each instrument. Harmonization of fluorescence intensity measurements between instruments has allowed for the development of one master analysis template to successfully generate matched exposure classifications on each instrument without the use of calibration curves or correction factors.

## Materials and methods

### Human blood sampling and irradiation

Peripheral whole blood samples from a total of 23 unique healthy adult human donors (recruited between October 12^th^ 2021 and March 20^th^ 2023, aged 21–63 years old, 12 females, 11 males, with no X-ray exposure in the past 6 months, written consent obtained, approved by Columbia IRB AAAS7621) were collected by venipuncture in BD Vacutainers^®^ with Sodium-Heparin (BD Biosciences 367878). At ~1–3 hours after collection, blood samples were mock or X-irradiated with 1, 2, 3, 4, or 5 Gy at CRR inside 50 mL conical tubes placed horizontally (with the blood spread into an even layer) on the shelf of an X-RAD 320 biological irradiator (Precision X-Ray Inc., North Branford, CT) operated at 320 kVp, 12.5 mA with a custom Thoreaus filter (1.5 mm Al, 0.25 mm Cu, 1.25 mm Sn,; HVL 4 mm Cu), and a Focus-to-Surface Distance (FSD) of 40 cm. The dose rate was 0.95 Gy/min. Dosimetry was performed prior to irradiating each batch of samples using an annually calibrated 10x6-6 ion chamber (Radcal, Monrovia, CA).

### Sample preparation: Culturing, staining, and fixation

All samples were prepared in triplicate at CRR as follows: 100 μL aliquots of mock or X-irradiated blood samples were cultured at 37°C, 5% CO_2_, for 2 days with 900 μL complete RPMI (15% FBS, 1% Pen-Strep) in Matrix^™^ 1.0 mL tubes (Thermo Fisher Scientific^™^ 3740TS, Waltham, MA). After culturing, peripheral whole blood aliquots were lysed for 10 minutes at room temperature with eBioscience^™^ 1X RBC Lysis Buffer (Invitrogen, 00-4333-57), washed in 1% BSA/PBS, and then fixed at 4°C using the Cytofix/Cytoperm^™^ Fixation/Permeabilization Solution Kit (BD Biosciences, 554714, Franklin Lakes, NJ). Fixed leukocyte cells were washed with perm/wash buffer from the Cytofix/Cytoperm^™^ Fixation/Permeabilization Solution Kit and then intracellularly stained with BAX-AlexaFluor488 (mouse, monoclonal-2D2, BioLegend 633604, 1:200) or phosphor-p53 Ser37 (rabbit, polyclonal, Cell Signaling Technology #9289, 1:200) for 1 hour in the dark, at room temperature, followed by a wash with perm/wash buffer. The p53 sample set was then stained with Alexa Fluor^™^ 488 (Goat anti-rabbit, Invitrogen A11034, 1:1000) for 1 hour in the dark, at room temperature. All samples were then washed twice with PBS and resuspended in 1 mL PBS until sample acquisition.

Fixed and stained samples were either stored at 4°C while protected from light at CRR or shipped to Health Canada in polystyrene boxes (Saf-T-Pak^®^ Inc. STP309) with a combination of chilled and frozen temperature packs (optimized to maintain a temperature range of 2–5°) and temperature loggers (EasyLog EL-USB-1). A standardized packing protocol was developed at CRR to maintain consistency between shipments.

### Imaging flow cytometry

#### Sample acquisition

Prior to acquisition, sample aliquots were centrifuged, and the fixed and stained cells were concentrated to a volume of 50 μL by removing the excess PBS. Samples were manually loaded and acquired on the ImageStreamX (ISX) MkII Imaging Flow Cytometers (Cytek, formerly Luminex Corporation, Austin, TX) at CRR (“CU-reference”), Columbia Stem Cell Initiative Flow Cytometry Core Facility (“CU-FlowCore”), and Health Canada (“HealthCanada”). The CU-reference instrument is equipped with one objective (40x), two lasers (488 and 785 nm), and one Charge-Coupled Device (CCD) camera, and the HealthCanada instrument is equipped with three objectives (20x, 40x, and 60x), four lasers (405, 488, 642 and 785 nm) and one CCD camera. The CU-FlowCore instrument is equipped similarly to the HealthCanada instrument, with an additional laser (561 nm) and CCD camera. The acquisition template originally designed at CRR for the CU-reference instrument was used to acquire samples on all three instruments, which inactivated the additional features on the CU-FlowCore and HealthCanada instruments that are not present or used on the CU-reference instrument. The acquisition template provided the following settings common across all instruments: low flow rate (0.5 x 0.5 μm^2^ pixel resolution, 10 μm core diameter, and 55 mm/second), 40× magnification, 3,000 focused and single cells collected using the same gating coordinates, 1 mW 785 nm laser, and 488 nm excitation laser settings on each instrument initially set to match the CU-reference instrument at 200 mW. Alexa Fluor^™^ 488 (AF488) or unstained signals were collected in channel 2 (480–560 nm filter), brightfield (BF) data was collected in channel 1 (430–480 nm filter), and side scatter (SSC) signal was collected in channel 6 (745-800nm filter). Acquired data files (.rif) were sent back to CRR for analysis.

#### Methods of instrument harmonization

**Fig 1:**
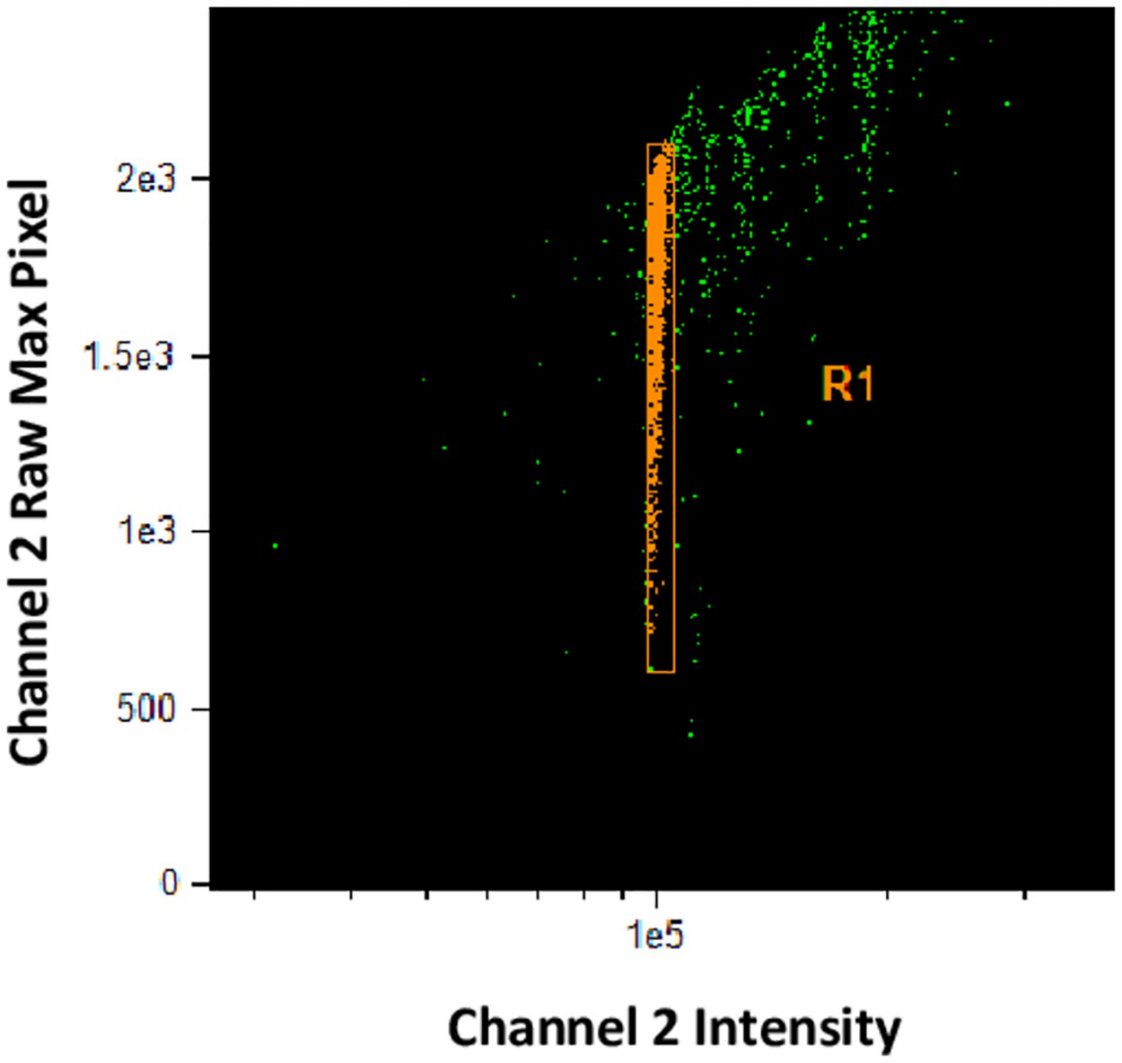
Harmonization of fluorescence intensity using rainbow beads. 488 nm laser power was modulated on each instrument while acquiring SPHERO^™^ Ultra Rainbow Quantitative Particle, Intensity 3 beads such that most of the single beads fell within region R1, with an MFI of 100,000.

In order to generate similar fluorescence signal captured on the CCD of each ISX MkII the 488nm excitation laser power was adjusted using one of the following 2 methods:

1. Samples were acquired simultaneously on each ISX MkII, and 488 nm excitation laser power on CU-FlowCore and HealthCanada instruments were adjusted so that mean values of the Intensity feature, (defined as the sum of the background subtracted pixel values within the masked area of the image signal) generated in Channel 2 (480–560 nm) were within ±5% of the mean Intensity values generated in Channel 2 on the CU-reference instrument.
2. Rainbow beads (SPHERO^™^ Ultra Rainbow Fluorescent Beads Intensity 3 from the Spherotech Ultra Rainbow Quantitative Particle Kit URQP-38-6K) were acquired on each ISX MkII instrument, and the 488 nm laser power on each instrument was adjusted so that the mean values of the Intensity feature generated by the rainbow beads in Channel 2 on each instrument were within ±0.5% of the Mean Fluorescence Intensity (MFI) target value of 100,000 (Fig 1).

#### Analysis: Population selection and biomarker quantification

The previously developed [8] analysis template using Image Data Exploration and Analysis Software (IDEAS^®^, Luminex ver. 6.2) was used in this study to select for leukocyte populations and define regions of biomarker measurements:

Focused cells were selected by visually inspecting captured cell images and using the BF gradient root mean square (“Gradient RMS”) feature. Single cells were gated using a bivariate plot of BF area versus BF aspect ratio, eliminating debris and doublets. For morphological selection of leukocyte subtypes, lymphocyte populations were gated using a bivariate plot of BF area versus intensity of the side scatter (SSC). Discrete event clusters with less BF area and SSC intensity were defined as “lymphocytes”.

To quantify non-specific background signal generated from each individual instrument, fluorescence intensity on the 480–560 nm detector (Channel 2) was examined in several unstained samples on each instrument and a mean “background” upper region boundary for each instrument was set in the gated lymphocyte population. All intensity values above this “background” upper region boundary were defined as “Positive”. To measure instrument-specific biomarker expression in the gated lymphocyte population, the uniform analysis template was applied to each biomarker-stained sample and automatically batch processed in IDEAS^®^. The MFI of the biomarker, and percentage of single cells that appeared in the “Positive” region (“% Positive”) of the biomarker fluorescence intensity were computed for samples generated on each instrument.

To remove the need for individualized analysis templates for samples generated on each ISX MkII, the harmonized sample measurements across the three instruments were used for the development of one master template for biomarker quantification, as follows: Fluorescence intensity on the 480–560 nm detector was examined in several unstained samples acquired from each of the three harmonized instruments, and mean x coordinates for the “background” region’s upper limit across the three instruments were computed and set for the region boundary. All intensity values above this master “background” region boundary were defined as “Positive”. For more details on the use of harmonized background values for the development of the master template, see Results section.

### Statistical analysis

Comparisons of MFI values between CU-FlowCore/HealthCanada instruments and the CU-reference instrument were analyzed by two-way ANOVA with Dunnet’s multiple comparison test. Comparisons of the differences of CU-FlowCore/HealthCanada MFI values from CU-reference MFI generated after both sample and bead harmonization methods were analyzed by Wilcoxon matched-pairs rank test with Holm-Sidaks correction for multiple comparisons. Linear associations between % Positive biomarkers measurements on CU-FlowCore/HealthCanada and the CU-reference instruments were evaluated by Deming Regression (with equal uncertainties on X and Y axes). Instrument bias and estimated intervals of agreement between % Positive biomarker measurements generated on the CU-FlowCore/HealthCanada and the CU-reference instruments were evaluated by Bland-Altman analysis. Receiver-Operator Characteristic (ROC) analysis was used to assess discrimination potential of % Positive biomarker measurements of triplicate samples acquired on each instrument based on exposure (unexposed = 0 Gy, exposed = 1–5 Gy). Two-tailed p-values less than 0.05 were considered statistically significant. All statistical analyses were performed using GraphPad Prism (version 10.0.0; GraphPad Software, Inc., La Jolla, CA).

## Results

### Comparison of baseline intensity values across three ISX MkII instruments

**Fig 2:**
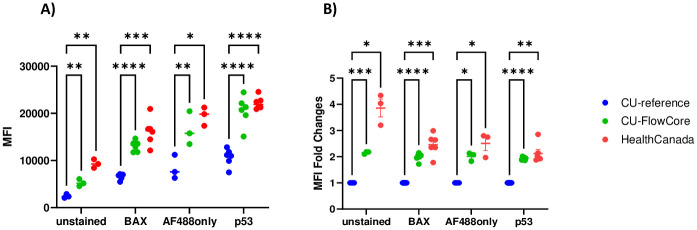
Interinstrument comparison of signal intensities. Triplicate samples with the denoted staining conditions were simultaneously acquired on IFC instruments at each site using the CU-reference instrument acquisition settings. A) Raw MFI values generated on each instrument. B) Fold changes of MFI values generated on each instrument. n = 3 and 6 for unstained/AF488 only and BAX/p53 stained samples, respectively; error bars represent ± SEM; *p < 0.05; **p < 0.01; ***p < 0.001; ****p < 0.0001; p-values reflect the significance of two-way ANOVA, with CU-FlowCore and HealthCanada means compared to the CU-reference instrument means.

To determine whether instrument harmonization would be necessary, we examined MFI of unirradiated samples generated from each instrument when all instruments used the same acquisition template from CRR, with matched settings (such as fluidic speed, camera settings, laser power, gating, and collection parameters). Fig 2 shows that in lymphocytes, fluorescent signal values detected on the CU-FlowCore and HealthCanada instruments were significantly higher (approximately 2-4-fold, p-values ranging < 0.05–0.001) than those generated on CU-reference instrument. Importantly, both conjugated (BAX) and unconjugated (p53) biomarker antibodies were tested, along with corresponding background controls (unstained and AF488 only, respectively), and this trend was observed in all staining conditions. These data suggest that instrument harmonization would be necessary to generate datasets compatible for analysis by one template.

### Instrument harmonization

Towards the development of one master template for the quantification of biomarker expression across instruments, we sought to harmonize the ISX MkIIs to generate similar MFI values using the following two reference materials:

(i) Samples: We simultaneously interrogated unstained samples at each site, and the laser powers of CU-FlowCore and HealthCanada instruments were adjusted to generate background signal intensities within ±5% of the CU-reference instrument. We also tested these adjusted laser settings on samples stained only with secondary-antibody (AF488) and found that these same laser powers also successfully generated values within ±5% of the CU-reference instrument.
(ii) Beads: We interrogated commercially produced polystyrene rainbow beads of uniform size with a standardized amount of embedded fluorophores and adjusted the laser powers of each instrument to generate intensities at the same target MFI value of 100,000.

**Fig 3:**
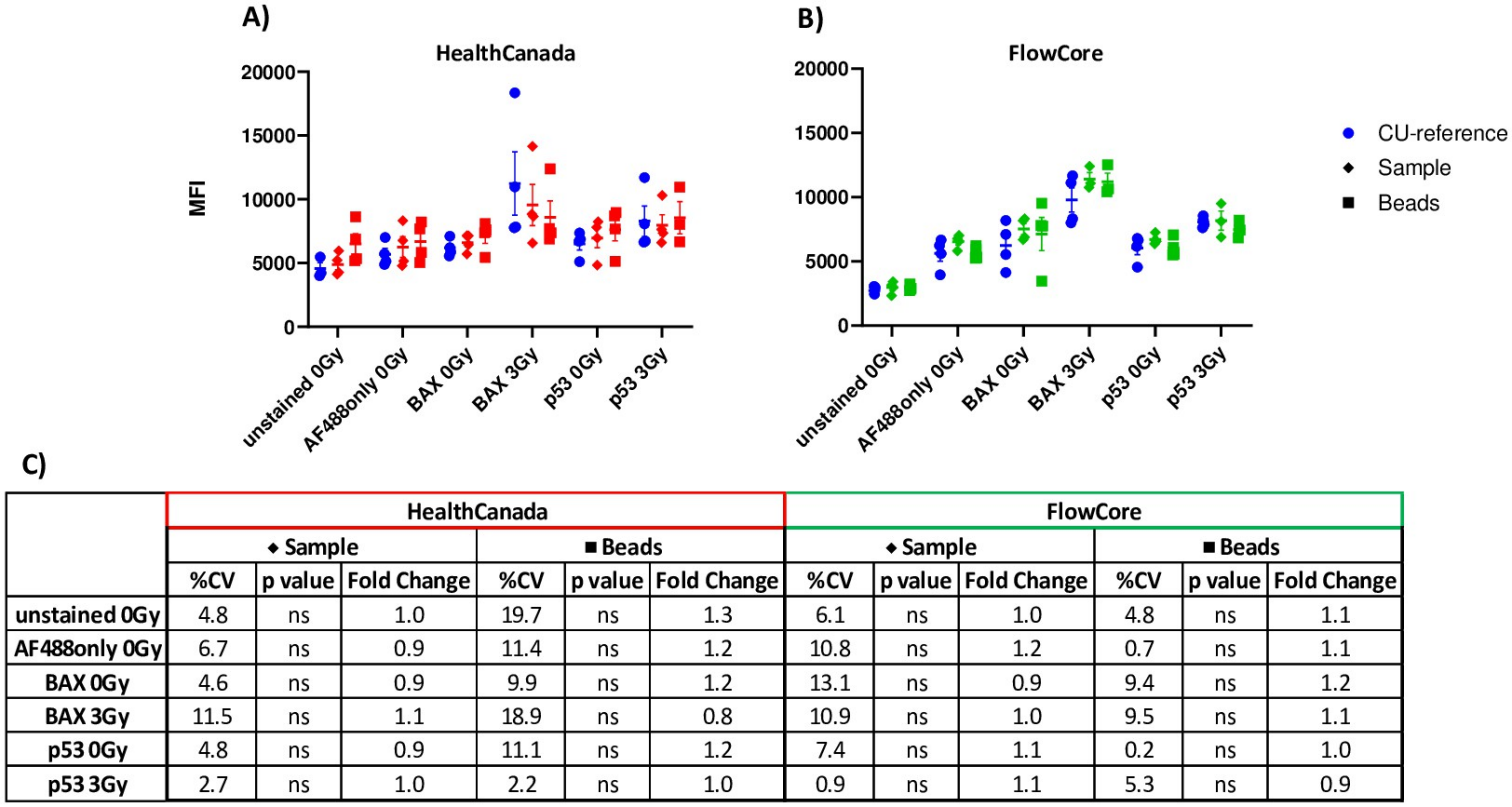
Harmonization of background and biomarker signal intensities using sample and beads methods at A) Health Canada and B) CU-Flow Core. Error bars represent ±SEM. C) Comparison table of intensity measurements generated on the HealthCanada and CU-FlowCore instruments after harmonization by beads and samples methods as compared to the CU-reference instrument. %CV values were calculated for harmonized MFI values on HealthCanada or CU-FlowCore and CU-reference instrument, in each test condition. p values reflect significance of two-way ANOVA, ns = not significant. Fold Change values for each replicate in each tested condition were calculated as (harmonized MFI value from HealthCanada or FlowCore instruments) / (MFI value from CU-reference instrument). n = 4.

After testing both harmonization methods, we compared MFI measurements of samples generated on either HealthCanada or CU-FlowCore instruments to those generated on the CU-reference instrument (Fig 3A and 3B; **related statistics and calculations shown in** 3C). These data show that no statistically significant differences were found between the MFI means of both instrument comparisons, and fold changes were successfully reduced to 0.8–1.3x, regardless of which harmonization method was used. We evaluated variability between MFI values generated on harmonized instruments compared to the CU-reference instrument and found CV values less than 15% between all harmonized values generated on HealthCanada or CU-FlowCore and CU-reference instrument, indicating a relatively low degree of variability between measurements generated on the reference instrument and those on instruments harmonized with either method. However, there is a higher degree of uncertainty remaining (approximately 19%) in the unstained and BAX 3 Gy samples after harmonization of HealthCanada instrument with beads.

We also compared efficacy of each harmonization method by examining the differences between harmonized and reference intensity values. S1 Fig shows that both methods of harmonization bring the intensity values generated on both instruments close to the CU-reference instrument values, and that the null hypothesis (that no statistical differences exist between the harmonization methods) can be accepted. These data suggest that both harmonization methods successfully improved the agreement in the output of all the instruments and generated MFI values within reasonable limits from the CU-reference instrument.

**Fig 4:**
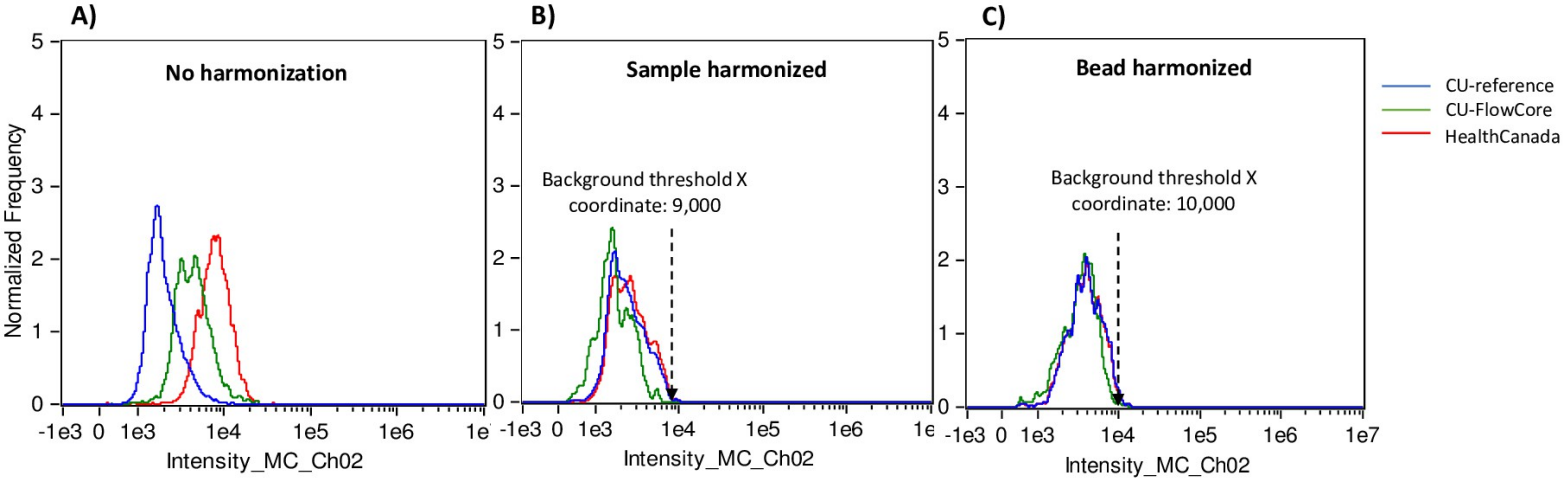
Representative histograms of spectra alignment pre- and post-harmonization. A set of triplicate unstained 0 Gy samples were acquired on each ISX instrument with either A) no harmonization (all samples acquired using the CU-reference instrument settings), B) sample harmonization, or C) bead harmonization. No common background threshold coordinate could be found between samples acquired on each ISX without harmonization, whereas a common threshold coordinate could be found after both sample and bead harmonization.

The harmonization of signal output across ISX MkII instruments allowed for the development of one master template for measuring % Positive biomarker responses across harmonized instruments. Fig 4 depicts a representative example of the emission spectra of triplicate unstained samples acquired on each of the three instruments. Alignment of the emission spectra generated on each harmonized instrument importantly created a common background threshold X-coordinate across the instruments. We thus examined the emission spectra of several unstained samples generated on all instruments and calculated the mean value of these common background threshold X-coordinates, which was then set as an assigned background threshold value in the master analysis template for quantifying % Positive biomarker responses across the three ISX Mk II instruments.

### Correlation and agreement of harmonized biomarker output across instruments

**Fig 5:**
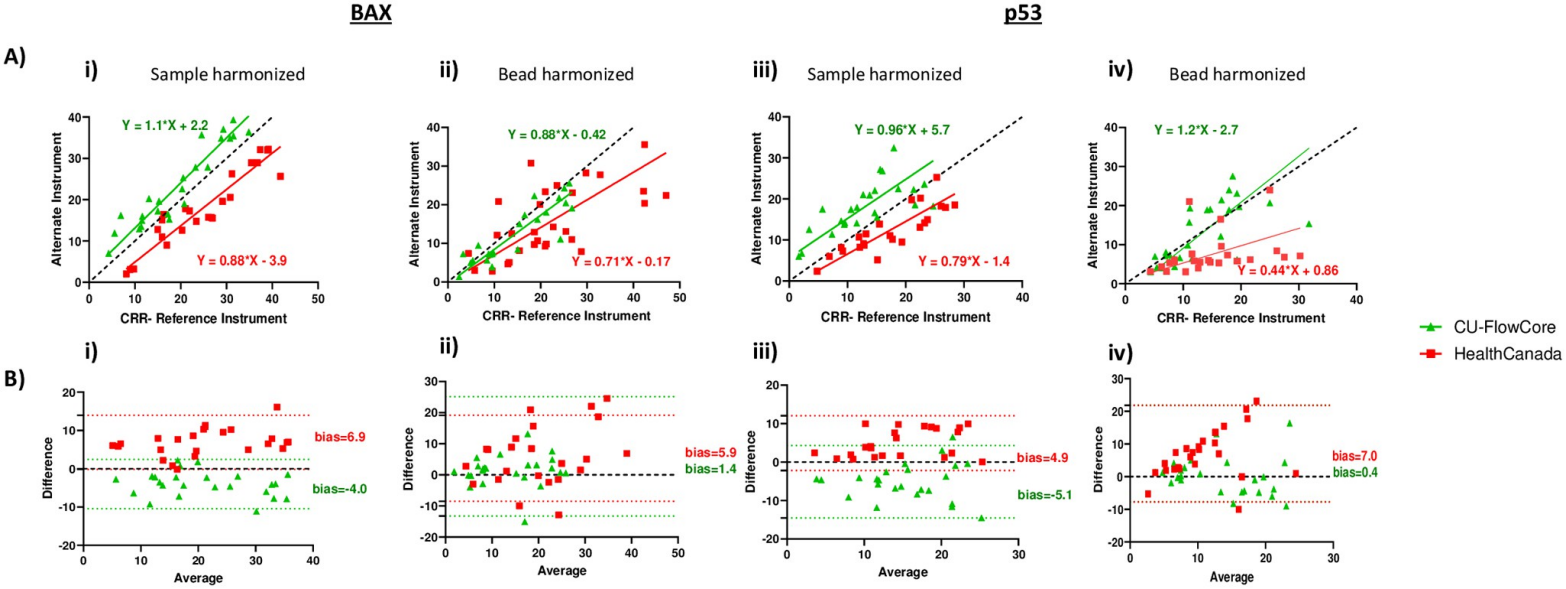
Correlation and agreement of harmonized biomarker measurements from triplicate 0–5 Gy irradiated samples acquired on alternate and reference instruments. A) Deming regression for evaluating correlation between the cells harmonized % Positive BAX and p53 measurements and reference measurements. Line of identity depicted as black dashed line. B) Bland-Altman plots for evaluating agreement between harmonized BAX and p53 measurements and reference measurements. 0% bias is depicted as dashed black line and limits of agreement are depicted as dotted colored lines. Difference = % Positive values from CRR Reference Instrument—% Positive values from alternate instrument. Average = Mean of CRR reference instrument and alternate instrument. Refer to figure key for interpretation of color. n = 9 (n = 4 and n = 5 for sample harmonized and bead harmonized datasets, respectively).

After harmonizing BAX and p53 MFI biomarker outputs on various ISX MkII instruments and developing the master analysis template, we sought to assess the strength of correlation and agreement of the harmonized % Positive values. In this study, 0–5 Gy X-irradiated triplicate samples were acquired simultaneously on HealthCanada, CU-FlowCore, and CU-reference instruments (these data can be found in S1 Table). To evaluate the association between harmonized biomarker measurements from the HealthCanada and CU-FlowCore instruments and the CU-reference instrument, Deming regressions were performed (Fig 5A; **Panels i to iv**). The data overall show strong correlations with CU-reference instrument measurements, with slopes generally ≥ 0.88 for both biomarkers. The HeathCanada sample harmonized p53 values (Fig 5Aiii) and bead harmonized BAX values (Fig 5Aii) showed slightly weaker correlations with CU-reference instruments with slopes of 0.79 and 0.71, respectively. The HealthCanada bead harmonized p53 values (Fig 5Aiv) show drastically weaker correlation with a slope of 0.44, potentially due to significant delays in sample shipping (3 days) as well as technical issues on the HealthCanada instrument following the arrival of the samples. In addition, these Deming regressions show that despite generally strong correlations, the intercepts deviate from the origin (-3.9 to 5.7), indicating various degrees of instrument bias. For both biomarkers, harmonization on both instruments generates values with lower % Positive values compared to the CU-reference instrument, with the exception of sample harmonization on CU-FlowCore which generated higher % Positive values.

In Fig 5B we also assessed the nature of measurement agreement by conducting Bland-Altman analysis to visualize systematic differences/error between the sample values. CU-FlowCore generally shows smaller bias than HealthCanada, ranging -4.0 to 0.4 versus 5.4 to 7.0, respectively, (aside from sample-harmonized p53 values where the biases are roughly equivalent to each other), indicating that CU-FlowCore generates values with lower average discrepancy from the CU-reference generated values. Overall, sample harmonized biomarker measurements show more narrow limits of agreements (Fig 5Bi and 5Biii, ranging 12.9 to 18.8) than bead harmonized biomarker measurements (Fig 5Bii and 5Biv, limits ranging 20.0 to 29.0). Examination of variability trends show that overall harmonized BAX and p53 values on both instruments display constant variabilities, indicating that the measured instrument bias remains consistent across the range of average biomarker values (and is not affected by the levels of biomarker output). However, bead harmonized p53 values on the HealthCanada instrument display a constant error, with differences between the methods and scatter around the bias lines both consistently increasing as the average values increase. This suggests that the HealthCanada instrument is more likely to underestimate the higher p53 values when harmonized by beads. Of note, the shipping delays and technical issues mentioned above in this sample batch may have contributed to these effects. Whether the discrepancies presented across all variables tested here (biomarker, instrument, harmonization method) are within acceptable limits will ultimately need to be determined by application to a diagnostic endpoint.

### Classification of radiation exposure

**Fig 6:**
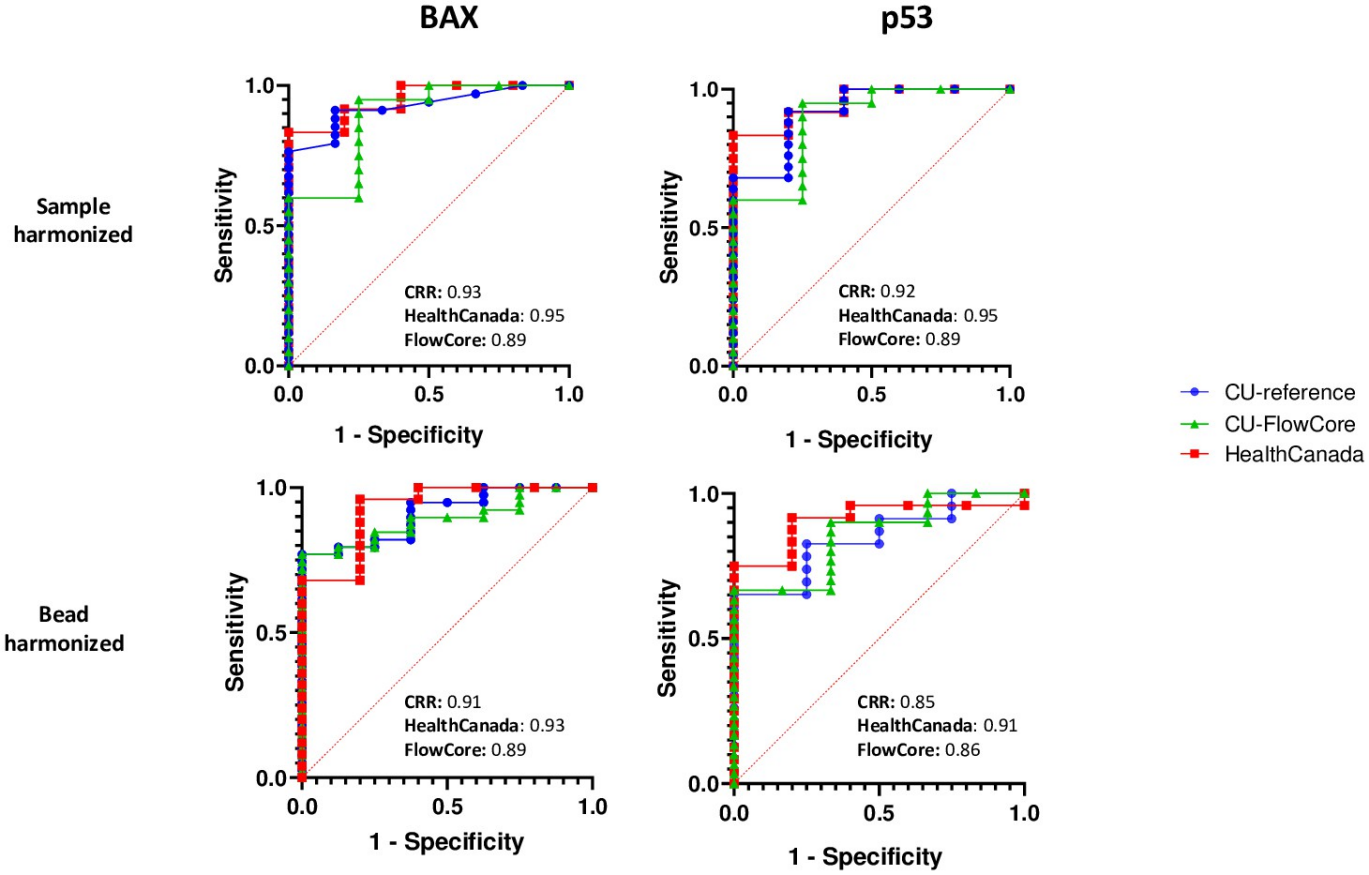
Radiation dose classification based on harmonized biomarker quantifications. ROC curve analysis to discriminate between unexposed (0 Gy) and exposed (1–5 Gy) was performed on % Positive biomarker quantifications generated from triplicate 0–5 Gy X-irradiated samples that were acquired at each site, harmonized by sample or bead protocols, and analyzed with one master template. Area under the curve (AUC) values for each ROC analysis are indicated in the figure. n = 9 (n = 4 and n = 5 for sample harmonized and bead harmonized datasets, respectively).

A major goal of this study was to use harmonized measurements from different ISX MkII instruments and one master analysis template to generate reproducible classifications of radiation exposure. To assess dose discriminating performance of the harmonized biomarker values using the 0–5 Gy X-irradiated triplicate samples that were acquired simultaneously on the CU-reference, CU-FlowCore and HealthCanada, instruments (previously shown in S1 Table, and Fig 5), we performed ROC curve analysis to distinguish between binary dose categories: unexposed (0 Gy) and exposed (1–5 Gy). Fig 6 shows that for both BAX and p53, calculated AUC values were ≥ 0.85 across triplicate samples acquired on each instrument, with both bead and sample harmonization methods. These data highlight that using one master analysis template, both biomarkers were able to successfully classify exposure status of a sample at any of the three tested sites, following either bead or sample harmonization protocols.

## Discussion

The development of a bioassay for early triage capable of accommodating a surge in sample volume following a wide-spread radiological incident requires reproducibility on multiple instruments. We present here an interlaboratory study to validate the reproducibility of two radio-responsive protein biomarkers in human peripheral blood for rapid radiation exposure classification using our previously developed IFC-based high-throughput assay [8]. Our goal here was first to test the variability of protein biomarker output across three ISX MkII instruments, located at Columbia and Health Canada. Given the known sources of variability in an interlaboratory study from sample preparation, the instrument, and analytical specificity [9], our study was designed with CRR as the central laboratory and reference instrument, keeping all aspects of assay protocol constant: blood samples, reagents, antibodies, acquisition templates, analysis templates and exposure classification. Due to unavoidable inherent variables in the lasers and optics on each ISX MkII which may lead to significant differences in the amount of fluorescence reaching each instrument’s detectors, it is not surprising that we found that matched samples generated different fluorescence intensity values on each ISX MkII (Fig 2). These differences in intensity measurements underscored the need for harmonization of radiation dose-dependent biomarker outputs generated on each ISX MkII instrument for successful exposure classification using one master analysis template.

In this work, we tested harmonization of fluorescence intensity values generated on ISX MkII instruments using two reference materials: blood samples prepared at CRR and commercially produced rainbow beads. While fluorescence intensity harmonization studies using VersaComp Antibody beads and rainbow beads have been previously conducted with conventional flow cytometers that use photomultiplier tube (PMT) detectors [10–12], to our knowledge, this study represents the first harmonization attempt on a cytometer using a Charged-Coupled device (CCD), such as an IFC which requires the adjustment of laser power to modulate the fluorescent signal intensity. Both sample and bead harmonization approaches successfully reduced the variability between the instruments (Fig 3), which resulted in matched diagnostic endpoints of exposure classification on each instrument (Fig 6), but they are not practically equivalent methods. Blood samples prepared in the laboratory are subject to inconsistencies due to factors such as interdonor variability, transportation, and technical handling of the samples, leading to fluctuations in target harmonization values and necessitating concurrent interlaboratory communication. By contrast, commercially produced rainbow beads possess no inherent variability, and a preassigned target value that remains constant between experiments allows for a fixed standard operating procedure (SOP) that does not require live communication between laboratories. Still, despite the dynamic nature of harmonizing with laboratory-created samples, we have demonstrated that samples can serve as a viable reference material and may be used in the event that commercially produced rainbow beads are not available.

Important to this work, we developed a master template for analyzing biomarker % Positive values generated from harmonized ISX instruments, removing the need for instrument-specific analysis templates and calibration curves. Subjective gating differences at each site may contribute to readout variability and the centralization of the data analysis with a master template removes individualized gating methods, which can cause interlaboratory variation [9,13]. Should samples be prepared and/or analyzed at other sites, a more complete validation of bioassay reproducibility and harmonization of other sources of variability will also be required. To this end, we have written an SOP for sample preparation which has undergone inter-user testing here at Columbia, laying the groundwork for future inter-laboratory testing of the sample preparation SOPs at various sites. Standardizing the laboratory-based sample preparation between sites will remove the bottleneck of sample processing at one site, ultimately increasing the capacity of this bioassay for large-scale triage purposes.

In this study, we evaluated the strength of association and concordance of % Positive biomarker values generated on each instrument after harmonization of acquisition settings, using one master analysis template. Although biomarker values on the HealthCanada and CU-FlowCore instruments showed generally good correlation with the values generated on CU-reference instrument (Fig 5A), Bland-Altman plots showed instrument bias, with generally constant variability throughout the averaged % Positive units (Fig 5B). However, whether these instrument discrepancies fall within acceptable limits of agreement is ultimately a clinical question, dependent upon diagnostic endpoint.

For the purposes of initial triage, we used the harmonized data and master analysis template for radiation exposure classification. The ROC curves on all instruments with either method of harmonization demonstrated a strong ability to classify whether or not a sample has been irradiated. This indicates that one processed blood sample may be sent to any of the three instruments harmonized in this study and matched ROC curve exposure classifications can successfully be generated with the use of one master analysis template (Fig 6). This reproducibility of exposure classification represents an important step for validation of this bioassay towards FDA clearance for rapid triage in large-scale radiological incidents [5]. Notably, even in the case of an experiment with low correlation, (see Fig 5Aiv/HealthCanada, which was likely due to the technical limitations in that sample batch), harmonization of fluorescence intensity output using the beads still successfully allowed for the alignment of signal spectra and the creation of one master analysis template to generate matched exposure classifications. Our successful harmonization approaches presented here provide the basis for additional studies to define the limits of acceptable measurement variability (as determined by diagnostic endpoint) and standardize harmonization methodology for further validation of biomarker reproducibility on multiple instruments.

In this work, we tested interlaboratory variability of intracellular protein biomarker expression and developed methods for instrument harmonization in a human *ex vivo* model, 2 days post-exposure. Dose-dependent increases in biomarker signal in the human *ex vivo* model were observed at Day 2 in our earlier work [8], and these conditions, which elicited a positive biomarker response, were used here for the purposes of developing the harmonization methodology for reproducible dose classification across instruments for the first time. Yet, it is important to consider that protein biomarker performance (and consequentially, biodosimetry results) may differ between different individuals across different variables such as such as time-points and dose ranges in both *ex vivo* and *in vivo* models. Together with studies aimed to examine biomarker performance across a range of variables which will determine the bioassay’s limitations and applications, we envision the future use of this harmonization methodology developed here for comprehensive validations of biomarker reproducibility on multiple instruments.

In summary, we have developed methods for harmonizing fluorescence intensity values of two separately labeled biomarkers generated by one fluorophore, and excited by one laser on multiple ISX MkII instruments. In the larger context of developing a bioassay capable of estimating radiation exposure from various predictors (dose rate, time after exposure, demographics), we envision multiplexing biomarkers for measuring simultaneous output from each immunolabeled lymphocyte. Expansion of this work in the future may incorporate additional lasers available for the ISX MkII instruments to provide feasibility of harmonizing co-labeled protein biomarkers towards a more comprehensive bioassay for radiation dose discrimination.

## Supporting information

S1 Fig Comparisons of raw intensity differences from CRR reference instrument, generated from beads and sample harmonization methods at A) Health Canada and B) Flow Core. MFI values from each harmonized sample (unstained and AF488only—0 Gy, as well as BAX and p53–0 and 3 Gy) were subtracted from values of corresponding replicate at CRR. n = 4; ns (not significant) reflects the significance of Wilcoxon matched-pairs signed rank test.
(TIF)

S1 Table Experimental values. Corresponding figures are listed on the leftmost column. N/A (not applicable) represents parameters not tested in the individual experiment; empty fields represent samples that were lost during sample acquisition.
(XLSX)
