# Supplementary figures and images for: Interlaboratory comparison of high-throughput protein biomarker assay quantifications for radiation exposure classification
Source: PLoS One. 2024 Apr 29;19(4):e0301418. doi: 10.1371/journal.pone.0301418 (PMC11057749; doi:10.1371/journal.pone.0301418)

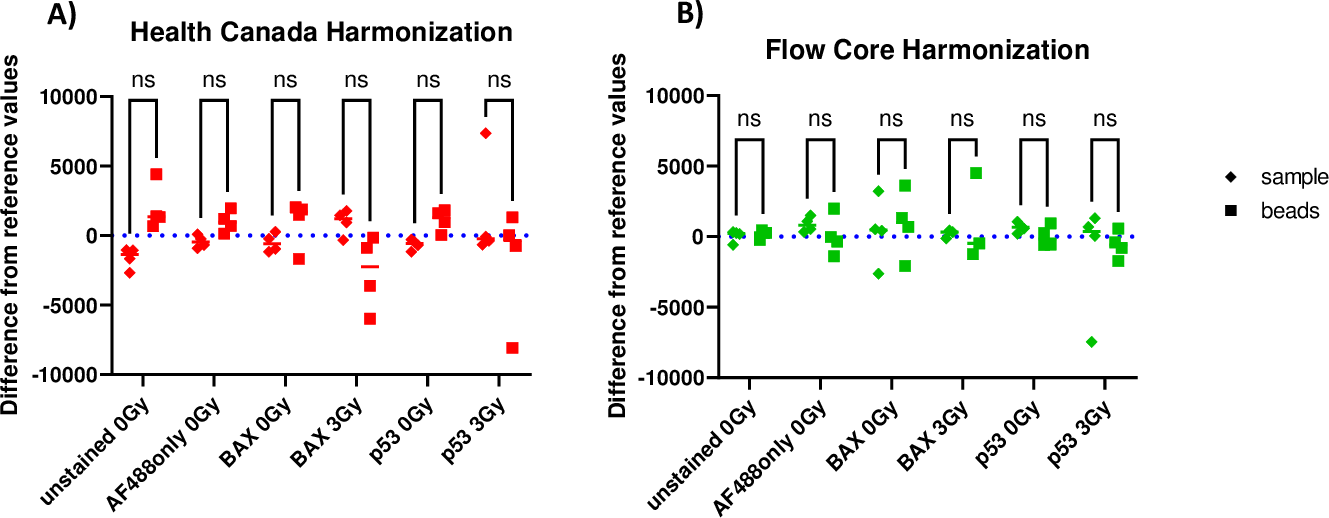

Supplement: S1 Fig — MFI values from each harmonized sample (unstained and AF488only—0 Gy, as well as BAX and p53–0 and 3 Gy) were subtracted from values of corresponding replicate at CRR. n = 4; ns (not significant) reflects the significance of Wilcoxon matched-pairs signed rank test. (TIF) [file pone.0301418.s001.tif]
